# Molecular and Immunological Characterization of *Staphylococcus aureus* in Pediatric Atopic Dermatitis: Implications for Prophylaxis and Clinical Management

**DOI:** 10.1155/2011/718708

**Published:** 2011-10-27

**Authors:** Chiara Pascolini, JoLinda Sinagra, Simone Pecetta, Valentina Bordignon, Alessandra De Santis, Laura Cilli, Viviana Cafiso, Grazia Prignano, Bruno Capitanio, Claudio Passariello, Stefania Stefani, Paola Cordiali-Fei, Fabrizio Ensoli

**Affiliations:** ^1^Clinical Pathology and Microbiology Laboratory and Pediatric Dermatology Division, San Gallicano Dermatology Institute, 00144 Rome, Italy; ^2^Department of Public Health and Infectious Diseases, University La Sapienza, 00185 Rome, Italy; ^3^Department of Microbiology, University of Catania, 95124 Catania, Italy

## Abstract

*S. aureus* represents a critical cofactor in atopic dermatitis (AD). In this paper, the prevalence of *S. aureus* infection/colonization was evaluated in 117 children as well as in their cohabitants, in order to assess the value of *S. aureus* characterization in predicting disease onset and severity and in providing indications for prophylaxis. Results showed that children with AD as well as their cohabitants had a significantly greater incidence of *S. aureus* infection/colonization as compared to controls. The genetic characterization showed a virtual identity of the bacteria strains collected at different sites of the patients with those found in the cohabitants, suggesting both a direct transmission between the nasal reservoir and the lesions in the same atopic subject and a risk for reinfection within family cohabitants. These data stress the need of preliminary laboratory assessment and posttherapy control in both AD patients and their close contacts for effective *S. aureus* eradication.

## 1. Introduction

Atopic dermatitis (AD) is an inflammatory disease affecting the skin and characterized by impaired epidermal barrier function and cutaneous inflammation [1, 2]. It is clinically characterized by an early onset, mostly occurring before 5 years of age [3]. The prevalence of AD has increased in industrialized countries during the past three decades, and among Italian schoolchildren the estimated prevalence is presently of about 5.8% [4]. The eczematous skin of the patients is highly susceptible to bacterial colonization, and *Staphylococcus aureus* (*S. aureus*) represents the most frequent isolate [5–8]. *S. aureus* is a Gram-positive opportunistic bacterium that, although part of the normal flora of the skin, can give rise, as a consequence of clonal evolution, to virulent strains characterized by the expression of virulence factors and the acquisition of resistance to a number of different drugs including *β*-lactams [9]. *S. aureus* infection appears to play an important role in the pathogenesis of AD by either causing or exacerbating skin inflammation [10–13]. In fact, *S. aureus* toxins have potent superantigenic properties (e.g., staphylococcal enterotoxin SEA, SEB, SEC, SED, and toxic shock syndrome toxin (TSST-1)) and may induce monocytes and lymphocytes activation, which in turn are induced to produce several inflammatory cytokines [14, 15]. In a healthy skin, this process causes an inflammatory reaction with a clinical outcome similar to that seen in AD [10]. 

Although the impact of *S. aureus* infection in the clinical manifestation of AD is still debated, antibiotic-corticosteroid combination therapy against *S. aureus* represents an important component of the therapeutic approach to AD [16] and appears effective against both the occurrence of secondary infections and the severity of the disease [17]. However, it should be also considered that the clinical benefits appear often transient and that the onset of an antibiotic resistance represents a serious emerging problem in long-term therapy [18, 19] suggesting the need of an accurate microbiological analysis for a most adequate and effective therapy.

In this study, we investigated the prevalence and relationship between *S. aureus *infection/colonization, in both skin lesions and anterior nares, and the clinical severity of the disease in children with AD as compared to a control group of asymptomatic children and examined the influence of *S. aureus *colonization in the family members as a potential source for reinfection. The results indicated that children with AD had a significantly greater prevalence of *S. aureus* infection/colonization as compared to controls. The genetic characterization confirmed the identity of bacteria strains collected from the patients with those found in the cohabitants, suggesting both a direct transmission between the lesions in the same atopic subject and a risk for reinfection within family cohabitants. These results emphasize the importance of a preliminary laboratory assessment and posttherapy control in both AD patients and their close contacts for effective *S. aureus* eradication. 

## 2. Patients and Methods

### 2.1. Patients and Controls

Patients incoming the Pediatric Outpatient Clinic at the San Gallicano Dermatology Institute were examined during a two-year period (2007-2008). One hundred and seventeen children aged between 3 months and 12 years (42 patients were under 12 months of age) of both sexes, suffering from mild to severe AD, were included in the study. Diagnosis of AD was based on the criteria described by Hanifin and Rajka [20]. The severity of AD was measured by the scoring atopic dermatitis index (SCORAD) of the European Task Force on atopic dermatitis. Briefly, a numeric score (1–10) was assigned to (a) extent and (b) intensity of skin lesions and (c) to subjective symptoms like pruritus and sleep loss. The final SCORAD was calculated by the following equation: a/5 + 7b/2 + c. In the present study, the clinical expression of disease was classified as low, medium, or high according to SCORAD values ranging between 0 and 15, 15, and 40 or >40, respectively. Patients did not receive any steroid or antibiotic therapy in the last two months before the initiation of the present study. Parents and cohabitant family members of AD children were asked to voluntarily participate to the study by allowing skin and nose sampling and examination aimed at *S. aureus* isolation and characterization. One hundred and ten subjects of 37 families accepted to enter the study. *S. aureus* strains were also examined in asymptomatic carriers consisting of 90 healthy children, of both sexes (39/51 M/F), aged between 3 months and 12 years and 80 adults, of both sexes, aged between 25 and 50, attending the Outpatient Dermatology Clinic to control pigmented lesions. All samples were obtained by sterile swabs, from skin and nose. All patients parents and healthy volunteers gave their written informed consent before study initiation.

### 2.2. Microbiological Analysis

Swabs were collected from lesions, normal skin areas as well as from nares of atopic children. Nasal swabs were performed in their cohabitants or control subjects. Samples were plated on enriched (blood agar), selective (Mannitol Salt Agar) and differential (MRSA ChromID-BioMérieux, France) media and incubated for 24–48 h at 37°C. Identification and antimicrobial susceptibility of *S. aureus* were performed by an automated diagnostic system (Card AST P580, Vitek 2, BioMérieux, France) and included the most frequently used drugs for therapeutic use against *S. aureus*. Bacteria isolates were classified as Methicillin-resistant *Staphylococcus aureus* (MRSA) on the basis of resistance to oxacillin (≥4 *μ*g/mL) and positivity of the Penicillin-Binding Protein latex agglutination test (PBP2', Oxoid, UK).

### 2.3. Molecular Characterization

Molecular analysis was performed through Multiplex-PCR as previously described [21] in order to assess the expression of the following genes: capsular antigen (*cap* 5-*cap* 8); agr-group (*agr)*; adhesins (*hl*s-*spa*-*ica* A-*atl*-*cna*-*sdr* E-*sdr* C-*fnb* A-*clf* A/B); toxins (*eta*-*sea*-*sej*-*sec*-*sed*-*sek*-*seq-tst-splB-lukE*); Panton-Valentine leukocydin-PVL (*lukS*/F). Biofilm production was assessed as previously described [22–24]. 

### 2.4. Immunoassay

To detect the production of staphylococcal enterotoxins AD and toxic shock syndrome toxin-1 (TSST-1) isolates were incubated in tryptone soya broth and brain heart infusion, respectively, for 24 h at 37°C. Supernatants were tested for the presence of exotoxins by two specific reverse passive latex agglutination commercial kits (SET-RPLA and TST-RPLA) according to the instructions of the manufacturer (Oxoid, UK). The sensitivity of these agglutination assays is 0.5 ng/mL (SET-RPLA) and 2 ng/mL (TST-RPLA), respectively.

### 2.5. PFGE Analysis

Genotyping of isolated *S. aureus *strains was performed by pulsed-field gel electrophoresis (PFGE) after DNA digestion with SmaI (Fermentas ER0661), following the CDC (Center of Disease Control and Prevention, USA) protocol [25]. Briefly, electrophoresis was performed with pulses ranging from 5 to 45 seconds at a voltage of 6V/cm at 14°C for 23 hours using the PFGE instrument CHEF DR II (Bio-Rad, USA). The gels were stained for 20 minutes with ethidium bromide solution and observed under UV illumination.

## 3. Statistical Analysis

The statistical treatment of the results gathered from the different groups of subjects as well as the different clinical outcomes was performed by applying contingency tables and Fisher's *t* test (between two groups) or *χ* ^2^ test (among three groups). The analysis has been performed by the GraphPad Prism version 5.00 for Windows (GraphPad Software, San Diego, Calif, USA).

## 4. Results

Of the 117 patients analyzed, 66 (57%) were found to harbor *S. aureus *(Table 1). In particular, we had positive isolation from skin lesions and nasal swabs in 47 patients (40.1%), while 19 patients (16.2%) had only nasal *S. aureus *colonization (Table 1). A prevalence of 36.4% of positive isolation were obtained from 31 family members of 20 patients, significantly higher (*P* = 0.0036) than the nonatopic adults examined as controls (Table 1). In healthy children, *S. aureus *was isolated at a significantly lower frequency (18 positive out of 90, 20%, *P* < 0.0001) from nasal swabs. Conversely, the frequency of detection from specimens collected from normal skin areas in patients with AD was low (4/117) and did not significantly differ from that of healthy children (0/90). Considering the age, the rate of positive isolation (including either skin or nares) was significantly higher in the group of children aged more than six (*P* = 0.0062) (Table 2). Regarding the impact on disease severity, the presence of *S. aureus *colonization appeared significantly related to a more severe clinical expression of pediatric AD (*P* = 0.0001) (Table 3). 

Taking into consideration the expression of virulence factors by *S. aureus* isolates, the prevalence of MRSA strains among the total number of individual isolates in atopic patients was 7.9%, being present in the nose (4.5%) and, more frequently, in the skin lesion (12.8%). According to the molecular analysis, *S. aureus *strains isolated from atopic children (a total of 119, of which 96 were from skin lesion and nares of the same patients, 19 from nares of skin negative patients and 4 from healthy skin areas of 4 skin and nare positive patients), 94 (79%) had one or more genes coding for virulence factors such as adhesins, enterotoxins, TSST-1, CAP 5/8, the PV leukocydin, or the capacity to form biofilm (Table 4). Significant associations were found between the severity of the disease and high biofilm producing strains or TSST-1 positivity (*P* = 0.0003 and *P* = 0.002, resp.) (Table 5). 

**Figure 1:**
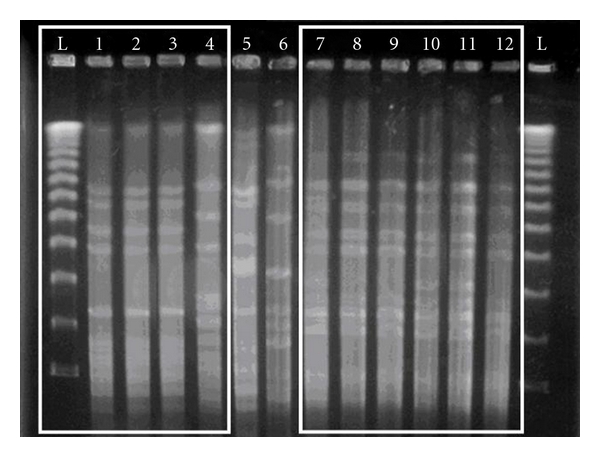
Pulse field gel electrophoresis (PFGE) of different isolates of *S. aureus;* the first and last lanes correspond to the DNA ladder; lanes 1–4 correspond to strains collected from lesional skin (1), nose (2), healthy skin (3) of an atopic patient (patient 16), and his father (4); lanes 7–12 show similar strains isolated from lesional skin, nose, healthy skin of an atopic patient (patient 26) and related mother, father, and brother.

To evaluate the possible transmission between nares and skin of the same patient or between patients and their cohabitants, the genetic characterization of strains was assessed by DNA restriction fragmentation by PFGE. Similar PFGE patterns were exhibited by strains collected from skin or nares in the same patient in all 47 positive cases, as reported in Figure 1. The PFGE analysis also showed a close similarity among patients and their cohabitants in 75% of cases. Interestingly, a striking correspondence was found between strains isolated from mothers and atopic children (72%), while fathers and brothers showed a lower extent of identity (53% and 58%, resp.). The data from DNA analysis were confirmed by bacterial phenotyping according to the profile of toxins released in the supernatants of *S. aureus* cultures as detected by the immunoassay (Table 6). The data showed a correlation between the individual patient and at least one of the family members. Considering the expression of drug resistance, the results indicated that strains isolated from parents had a more complex profile of drug resistance than the similar strains isolated from their children (Table 6). 

## 5. Discussion

Although the etiology of atopic eczema remains unknown, evidence suggests that both genetic and environmental factors play a role in determining both the susceptibility and the severity of the disease [5, 26]. Recent evidence suggests that *S. aureus* may play a key role in the pathogenesis of AD [8]. In fact, while *S. aureus *is rarely found on the skin of healthy subjects, as confirmed by our data on 80 control children, is very frequently found in patients suffering of atopic eczema. Several possible pathogenic mechanisms have been suggested for the role of *S. aureus *infection/colonization in AD pathogenesis pointing at either a direct chemical irritation or a nonspecific reaction of the *S. aureus* protein A with immune cells [27] as well as possible mechanisms involving superantigens production [28], which, in turn, exacerbates or maintain skin inflammation in atopic eczema. Indeed, *S. aureus* produces a group of toxins, which are capable of stimulating large populations of T-lymphocytes, even at distance from the eczematous sites, thus sustaining the activation of the immune system and the persistence of existing lesions. Conversely, the use of oral antibiotics as well as of topical antibiotics/antiseptics in combination with a topical steroid, antifungal agents or antiseptic bath additives, are often effective for the clinical management of AD and help reduce the severity of eczema and at improving the quality of life. 

In the present study, we characterized the strains of *S. aureus* isolated from skin lesion and anterior nares of children with atopic dermatitis and from their cohabitants. The same analysis was performed in healthy subjects either children or adults. Our findings showed that the incidence of *S. aureus* infection/colonization is higher in children with AD, as compared to matched healthy controls (*P* < 0.0001) (Table 1). These data are in agreement with the prevalence previously found by others (50%−64.2%) [29, 30]. Interestingly, the prevalence of* S. aureus* colonization was higher in the patient's relatives (36%) (*P* = 0.0036) than that found in adult healthy subjects (Table 1). In fact, *S. aureus* isolates obtained from different sites of the same patients had a close biologic and molecular identity with those collected from patients' cohabitants (*P* < 0.0001), indicating that they harbored the same *S. aureus* strain. A reason for such a high rate of *S. aureus* colonization among the cohabitants of pediatric patients should be probably due to the close physical contact occurring between children and parents/relatives, especially required for care giving. This may concur in establishing a source for reinfection among family members as well as other cohabitants and may sustain intrafamiliar spreading with persistence or reactivation of the disease [30–32]. Considering the expression of drug resistance, results showed in Table 6 indicated that strains isolated from parents had a more complex profile of drug resistance than the similar strains isolated from their children. This could be probably due to an increased exposure to antibiotics given both systemically and locally in adults during their lives. On the other hand, we also found that the presence of *S. aureus* was associated with an age under three yrs or more than six yrs (*P* = 0.0062) (Table 2), and with a more marked severity of the disease (Table 3). It could be hypothesized that the exposure during the neonatal period is due to the close contact with their caregivers, while older children more frequently interact with the external environment. The assessment of the bacterial genotypic and phenotypic profiles, through immunologic and molecular methods, showed a significant higher frequency of *S. aureus* strains expressing molecules associated with virulence, such as enterotoxins, adhesins, PVL, biofilm production, or MRSA in children with AD as compared to asymptomatic carriers. Among these biologic properties, the ability to generate biofilm or TSST-1 were significantly more frequent (*P* = 0.0003 and *P* = 0.002, resp.) in patients with a more severe form of the disease as compared to those with a mild disease expression according to the criteria described by Hanifin and Rajka [20]. These factors may thus have an important impact on the clinical outcome and disease severity. 

## 6. Conclusions

Our data suggest that the nasal cavity represent a key source for* S. aureus* skin colonization and that the familiar environment may play an important role in *S. aureus* colonization. Therefore, the eradication of these reservoirs might have a key impact on both the frequency and outcome of AD [33]. These results may indicate that clinical management of AD should include a routine testing for *S. aureus *not only on skin lesion but also in other important body reservoirs such as the nasal cavity [31] and emphasizes the need of performing susceptibility tests for antimicrobial drugs before initiating the therapy. In addition, routine testing should include also the family members of AD patients in order to break the chain of transmission through the establishment of an appropriate antibacterial therapy. 

## Figures and Tables

**Table 1:** Prevalence of *S. aureus* colonization in patients, family members, and controls. Contingency analysis and Fisher's exact test: *P < 0.0001; **P = 0.0036.

Subjects	Pos/all	Lesional skin and nares	Nares	Uninvolved skin
Atopic children	66/117* (57%)	47/117 (40.2%)	19/117 (16.2%)	4/117 (3.4%)
Healthy children	18/90 (20%)	—	18/90	0/90
Parents/relatives	31/85** (36.4%)	—	31/85	0/85
Healthy adults	16/80 (20%)	—	16/80	0/80

**Table 2:** Prevalence of *S. aureus *isolates in children with AD according to age.

Age (yrs)	Pos/neg (n)	Positive (%)
<3	27/22	54.1
3–6	13/21	38.2
6–12	26/8	75.7
*Chi-square test *P* = 0.0062.		

**Table 3:** Prevalence of* S. aureus *isolates in children with AD according to disease severity.

Disease score	Pos/neg	Positive (%)
Low	3/17	15.0
Medium	25/23	52.0
High	38/11	77.5
*Chi-square test *P* = 0.0001		

**Table 4:** Prevalence of *S. aureus *toxigenic strains in atopic and healthy children.

	Toxigenic strains Pos/all (%)	MRSA Positive (%)
AD lesional skin (47)	37 (79%)	6 (12.8)
AD uninvolved skin (4)	3 (75%)	
AD nares (66)	51 (77%)	3 (4.5)
CTRLs nares (18)	5 (28%)*	0/18
*Chi-square test *P < 0.0001		

**Table 5:** Molecular characterization of bacterial virulence factors of *S. aureus* strains isolated in children with AD and disease severity.

Disease Score	Biofilm	Adhesins	Enterotoxins	PVL	TSST-1	CAP 5/8
	Low/High	Pos/Neg	Pos/Neg	Pos/Neg	Pos/Neg	Pos/Neg
Low/Medium	19/15	32/0	26/6	1/31	12/20	34/0
High	8/43	58/0	45/13	1/57	28/31	51/0
Fisher's *t* test	*P* = 0.0003		*P* = 0.63	*P* = 0.18	*P* = 0.002	

**Table 6:** Immunological detection of toxin array and drug resistance of *S. aureus* isolates from patients and their family members.

Source of isolate	Localization	Enterotoxins TSST-1	Antibiotic resistance
Patient 6	Lesional skin	SEC	GM, P
	Nose	SEC	P
Patient 6, father	Nose	SEC	P
Patient 6, mother	Nose	SEC	P

Patient 10	Lesional skin	SEC	P
Patient 10, father	Nose	SEC	P

Patient 15	Lesional skin	SEB	OX, P, E
	Nose	SEB	P
	Healthy skin	SEB	OX, CM, MUP, RF
Patient 15, mother	Nose	SEB	P, CM,

Patient 16	Lesional skin	SEA, TSST-1	P, E, MUP
	Nose	SEA, TSST-1	P, E, MUP
	Healthy skin	SEA, TSST-1	P, E, MUP
Patient 16, father	Nose	SEA, TSST-1	OX, P, E, MUP, MXF,
			CM, TEC, TE, FA

Patient 19	Nose		P
Patient 19, mother	Nose		P

Patient 21	Lesional skin		FF
	Nose		Te
Patient 21, father	Nose	SEA, SED	P
Patient 21, mother	Nose	SEA, SED	P

Patient 23	Lesional skin		P
	Nose		P
Patient 23, father	Nose		P, CM, E
Patient 23, mother	Nose		P

Patient 25	Lesional skin		
Patient 25, mother	Nose		

Patient 26	Lesional skin		P, FF
	Nose	TSST-1	P
	Healthy skin		P, FF
Patient 26, father	Nose		P, FF
Patient 26, mother	Nose	TSST-1	P, E, CM
Patient 26, brother	Nose	TSST-1	P, E, CM

Patient 27	Lesional skin	SEB	P, E, CM
	Nose	SEB	P, E, CM
Patient 27, mother	Nose	SEB	P, E, CM
Patient 27, brother	Nose	SEB	P, E, CM

Patient 28	Lesional skin		
Patient 28, father	Nose		P, MUP
Patient 28, mother	Nose	TSST-1	

Patient 29	Lesional skin	TSST-1	P
Patient 29, mother	Nose	TSST-1	P

Patient 30	Lesional skin	TSST-1	P
	Nose		P
Patient 30, mother	Nose	TSST-1	P

Patient 31	Nose	SEC	P, E, CM
Patient 31, mother	Nose	SEC	P, E, CM

Patient 32	Nose		
Patient 32, father	Nose	TSST-1	P
Patient 32, mother	Nose	TSST-1	P
Patient 33	Nose	TSST-1	P
Patient 33, mother	Nose	TSST-1	P

Patient 38	Lesional skin		P
	Nose		P
Patient 38, father	Nose		P
Patient 38, sister	Nose		P

Patient 44	Lesional skin	SEC	P, E, CM, FF
	Nose	SEC	P, E, CM
	Healthy skin	SEC	P, E, CM
Patient 44, father	Nose	SEC	
Patient 44, mother	Nose	SEC	P, E, CM

Patient 45	Nose	TSST-1	P
Patient 45, mother	Nose	TSST-1	P

Patient 58	Lesional skin		P
	Nose		P
Patient 58, father	Nose		P
Patient 58, sister	Nose		P

Abbreviations: P: Benzylpenicillin; OX: Oxacillin; CM: Clindamycin; E: Erythromycin; FF: Fosfomycin and nitrofurantoin; FA: Fusidic Acid; GM: Gentamicin; MXF: Moxifloxacin; MUP: Mupirocin; RF: Rifampicin; TEC: Teicoplanin; TE: Tetracycline.
